# Uncovering the hidden mutational landscape of the oral epithelium

**DOI:** 10.1038/s41432-026-01224-0

**Published:** 2026-05-21

**Authors:** Manas Dave

**Affiliations:** The University of Manchester, Manchester, UK

**Keywords:** Anatomy, Oral anatomy

## Abstract

**A Commentary on:**

**Lawson ARJ, et al**.

Somatic mutation and selection at the population scale. Nature. 2025;647(8089):411–420. https://doi.org/10.1038/s41586-025-09584-w.

**Design:**

A cross-sectional observational cohort study using advanced genomic sequencing to map somatic mutations and clonal selection in normal human tissues at the population level^1^.

**Participants:**

The authors used targeted NanoSeq, a duplex sequencing method achieving exceptionally low error rates (fewer than five errors per billion base pairs). The technique was applied to targeted capture of a 239-gene panel (0.9 Mb) in 1042 non-invasive buccal swab samples (median donor age 68 years, 79% female, 37% smokers) and 371 blood samples from the TwinsUK registry.

**Data analysis:**

Mutation calling required strict duplex consensus filters. Gene-level selection was quantified using dNdScv, while site-level dN/dS analyses were used to identify recurrent hotspots and selected sites. Mutational signatures were deconvolved with sigfit, and multivariate mixed-effects regression modelled associations with age, smoking (pack-years), alcohol (drink-years), missing teeth, and other covariates.

**Results:**

In the oral epithelium, single nucleotide variants (SNVs) accumulated at 18.0 per cell per year and indels (insertions or deletions in the genome) at 2.0 per cell per year. Across all 1,042 participants, the authors detected 341,682 somatic mutations, including approximately 62,000 estimated driver mutations across 46 positively selected genes (e.g., NOTCH1 mutant cells reaching approximately 10% by ages 65–85 years, TP53 ~ 3%). Nine genes showed negative selection. Two dominant mutational signatures emerged: a clock-like signature A (resembling SBS1 and SBS5) and an alcohol associated signature B (resembling SBS16). The authors reported that smoking and alcohol were associated with higher mutation burdens, and that some effects on clonal selection were inferred from regression analyses; missing teeth (a marker of poor oral health) were independently associated with higher overall driver density. Driver frequencies for NOTCH1 were similar in normal epithelium and head and neck squamous cell carcinoma (HNSCC), whereas TP53 was enriched in cancer.

**Conclusions:**

The authors concluded that optimised NanoSeq enables population-scale mutational epidemiology in polyclonal tissues, revealing an extraordinarily rich landscape of positive and negative selection in normal oral epithelium. Environmental exposures shape mutagenesis and may also influence clonal selection, supporting a plausible mechanistic link between smoking, alcohol, and oral cancer risk.

## GRADE Rating:



## Commentary

The progression of oral squamous cell carcinoma (OSCC) is classically understood as a multi-step process, often preceded by clinically visible oral potentially malignant disorders (OPMDs) such as leukoplakia. However, the molecular foundations of this progression begin long before any phenotypic changes are visible. Historically, profiling somatic mutations in normal, healthy tissue has been severely restricted by the inherent error rates of standard next-generation sequencing.

In this landmark paper, the authors addressed this fundamental barrier by using a highly sensitive duplex sequencing method, targeted NanoSeq^1^. By examining 1042 buccal swabs, the study presents an unprecedented population-scale map of somatic mutations in the normal oral epithelium.

For the dental research community, this study is highly important. Firstly, it shows that buccal swabs can be used for large-scale molecular epidemiology of the oral epithelium. Secondly, it characterises the normal oral mucosa not as a static tissue, but as a tissue composed of many small clones under detectable selection. The authors identified 46 genes that were under positive selection in the sampled oral epithelium. Previous research has shown a high burden of somatic mutations and positive selection in normal skin^2^, but this scale of characterisation in the oral cavity has not been seen before.

Furthermore, the study shows that classical oral cancer risk factors such as age, tobacco smoking, and alcohol consumption are associated with mutation burden and inferred clonal selection. While the epidemiological link between these factors and oral cancer has been shown before^3^, the authors of this study provide high-resolution, *in vivo* quantification of their mutational impact at the single molecule level within sampled oral epithelium from a population cohort. The ability to track early clonal expansions non-invasively provides the framework for substantial future oral research.

The limitations of this study include an enriched cohort of older adults, predominantly female participants, self-reported exposure data including an extrapolated drink-years metric, limited HPV information, and the absence of a prospectively collected OPMD or OSCC comparator cohort analysed with the same pipeline, although comparisons were made with existing HNSCC datasets.

Practice points

- Age, tobacco smoking, and alcohol exposure are associated with higher somatic mutation burdens in clinically normal oral epithelium, reinforcing their established importance in oral cancer prevention.
- Normal oral mucosa is not genetically static; it contains numerous small clones under positive selection, indicating that molecular alterations can precede visible clinical disease.
- Non-invasive buccal swab sampling combined with high-accuracy NanoSeq provides a promising platform for future molecular epidemiology of the oral epithelium.
